# Dr. Clara E. Bowen

**Published:** 1908-02

**Authors:** 


Dr. Clara E. Bowen, of Buffalo, died at the German Hospital, December 28, 1907, aged 47 years. She graduated from the medical department, University of Buffalo, with the class of 1892. She began practice soon afterward and for many years had lived at 1223 Lovejoy Street, where she had a large clientele. A surgical operation was found necessary at the hospital which proved unavailing and she died soon afterward. Funeral services, conducted by the Rev. L. A. Wright, pastor of the Lovejoy Street Methodist Episcopal Church, were held at her home Monday, December 30. The remains were transported to Keene, N. H., for burial.

